# Effects of *Guanxinshutong* Capsules as Complementary Treatment in Patients With Chronic Heart Failure: Study Protocol for a Randomized Controlled Trial

**DOI:** 10.3389/fphar.2020.571106

**Published:** 2021-01-14

**Authors:** Yu Wang, Jiaping Xu, Jiehong Yang, Ling Zhang, Yuanjiang Pan, Liping Dou, Peng Zhou, Yizhou Xu, Chang Li, Yu He, Huifen Zhou, Li Yu, Jingwen Chen, Shuwei Huang, Wei Fu, Haitong Wan

**Affiliations:** ^1^ Institute of Cardio-cerebrovascular Disease, Zhejiang Chinese Medical University, Hangzhou, China; ^2^ School of Basic Medicine and Public Health, Zhejiang Chinese Medical University, Hangzhou, China; ^3^ Department of Chemistry, Zhejiang University, Hangzhou, China; ^4^ Department of Cardiology, Second Affiliated Hospital of Zhejiang Chinese Medical University, Hangzhou, China; ^5^ Institute of Brain and Heart CO Treatment, Zhejiang Chinese Medical University, Hangzhou, China; ^6^ Department of Cardiology, Hangzhou First People’s Hospital, Hangzhou, China; ^7^ School of Pharmacy, Zhejiang Chinese Medical University, Hangzhou, China; ^8^ Department of Cardiac-Cerebral Diseases, Yinchuan Cardiac-Cerebral Treatment Internet Hospital, Yinchuan, China

**Keywords:** Guanxinshutong (GXST), traditional Chinese medicine (TCM), heart failure, complementary medicine, clinical trial

## Abstract

Chronic heart failure (CHF) is a common cardiovascular disease with high mortality and a poor prognosis, which places heavy burdens upon society and families. Traditional Chinese medicine (TCM) has been used extensively as complementary treatment for CHF. *Guanxinshutong* (GXST) capsules are used commonly for the treatment of coronary heart disease (CHD). Experimental research and small-sample clinical trials have shown that GXST can attenuate CHF. However, the effects of GXST as complementary medicine in CHF treatment lack high-quality clinical evidence. We have designed a multicenter, randomized, double-blind, placebo-controlled clinical trial that explores the efficacy and safety of using GXST compared with placebo for patients with CHF with reduced left ventricular ejection fraction (LVEF). A total of 480 participants will be assigned randomly to the GXST group or placebo group at a 2:1 ratio. GXST and placebo will be added to standard treatment for 12 weeks, and then followed up for another 40 weeks. The primary outcome is the improvement value of 6-min walk distance, and the secondary outcomes include plasma levels of N-terminal pro-B-type natriuretic peptide, New York Heart Association classification, Minnesota Living with Heart Failure Questionnaire scores, echocardiographic parameters, and clinical endpoint events. Adverse events will be monitored throughout the trial. Data will be analyzed following a predefined statistical analysis plan. This study will show the effects of the specific use of GXST in CHF patients with reduced LVEF. The Research Ethics Committee of the Second Affiliated Hospital of Zhejiang Chinese Medical University has approved this study (2019-Y-003-02). Written informed consent of patients will be required. This trial is registered in the Chinese Clinical Trial Registry (ChiCTR1900023877). Our results will be disseminated to the public through peer-reviewed journals, academic conferences, and the Internet.

## Introduction

Chronic heart failure (CHF) is a complex clinical syndrome characterized by reduced cardiac output, insufficient organ perfusion, and venous congestion due to cardiac dysfunction (Coronel et al., 2001; Tan et al., 2010). CHF affects about 26 million people worldwide, with more than one million people hospitalized in the USA and Europe each year (Ambrosy et al., 2014). In China, it is estimated that 4.5 million aged 35–74 years suffer from CHF, and, with an increasingly aging population, the incidence of common cardiovascular diseases, such as hypertension, coronary heart disease (CHD), and CHF, continues to rise (Guo et al., 2003; Huo et al., 2019). CHF brings a heavy economic burden to society, families, and individuals, so new therapies and prevention strategies are needed.

According to guidelines for the diagnosis and treatment of heart failure set by the Chinese government in 2018, angiotensin-converting enzyme inhibitors (ACEIs), β-receptor blockers, and aldosterone receptor antagonists are recommended as “the golden triangle treatment” for patients suffering from CHF with reduced ejection fraction (Heart Failure Group of Chinese Society of Cardiology and Editorial Board of Chinese Journal of Cardiology, 2018). Although the use of the golden triangle treatment has improved the curative effect, this treatment plan is associated with hyperkalemia, deterioration of renal function (Heart Failure Group of Chinese Society of Cardiology and Editorial Board of Chinese Journal of Cardiology, 2018), limited improvement of symptoms, and unsatisfactory improvement in long-term survival (Pitt et al., 2014; Maisel et al., 2018). In recent years, the use of angiotensin receptor neprilysin inhibitors (ARNIs) has been considered as a therapeutic strategy in heart failure (McMurray et al., 2014). The PARADIGM-HF trial provided evidence that combined inhibition of the angiotensin receptor and neprilysin is superior to inhibition of the renin–angiotensin system alone in patients with CHF (McMurray et al., 2014). However, the ARNIs (tablets of sacubitril valsartan sodium) are not widely used in China currently.

Traditional Chinese medicine (TCM) is a popular type of supplementary and complementary medicine. TCM formulations have been employed widely and successfully for CHF treatment in China for more than 2000 years. Currently, the integration of Chinese and Western medicine has shown promising benefits in controlling symptoms, reducing mortality, improving cardiac function, and promoting quality of life in patients with CHF (Xu and Xu, 2011). In TCM, all the related symptoms, signs, tongue appearances, and pulse feelings at a certain stage of disease are summarized as a syndrome (“*Zheng*” in TCM) (Jiang et al., 2012). The syndrome is not only the core of TCM theory but also the base of the definitive diagnosis and efficacious therapies (Xu and Chen, 2008). According to Cui and colleagues, the basic syndrome type of CHF is “blood stasis syndrome” (Cui et al., 2009). The symptoms of CHF with blood stasis syndrome are palpitation, pain in the chest and hypochondrium, prominent blue veins in the neck, dark and purple complexion, cyanotic lips and nails, and edema of the lower extremities combined with a purple tongue, knotted/intermittent/uneven pulses.

*Guanxinshutong* (GXST) capsules have been developed by Shaanxi Buchang Pharmaceutical Co., Ltd. (Shaanxi, China) and gained approval by the China Food and Drug Administration (CFDA) for CHD treatment (approval number: Z20020055) in 2002. GXST consists of five herbal medicines (Table 1), and promotes blood circulation, removes blood stasis, and relieves pain (Zhang et al., 2016). Experimental studies have shown that GXST can inhibit ventricular remodeling through increasing mitochondrial productivity (Zhang et al., 2015; Zhang et al., 2016), repressing the expression of matrix metalloprotein-9, angiotensin receptors-1 and extracellular regulated protein kinases-2 (Zhang et al., 2012; Zhang et al., 2013), and the transforming growth factor-β/Smad signaling pathway (Fang et al., 2019) in myocardial tissue to improve CHF. A clinical trial with 61 patients reported that, compared with conventional treatment, combination with GXST could significantly increase left ventricular ejection fraction (LVEF) and cardiac output, and reduce the end-systolic, diastolic volume in patients with acute myocardial infarction with CHF (Li, 2019). Meanwhile, in our previous clinical study, GXST exerted a therapeutic effect on CHF (unpublished data), which implied that it might be a potential Chinese-patent medicine for CHF treatment. However, the current clinical data of GXST as a complementary therapy in CHF are lack of high quality, and limited in terms of methodology and sample size. Therefore, we designed a large-scale, multicenter, double-blinded clinical trial to investigate the efficacy and safety of GXST in CHF patients with reduced LVEF. The primary hypothesis of this study is as follows: combined with routine standard treatment, GXST is superior to placebo in patients with reduced LVEF caused by CHD.

**TABLE 1:** Components of GXST (intervention drug).

Scientific name	Chinese Pinyin	Latin scientific name	Parts & form used
*Choerospondias axillaris* (Roxb.) B. L. Burtt & A.W. Hill.	Guang Zao	*Choerospondiatis Fructus*	Dried ripe fruit
*Salvia miltiorrhiza* Bunge	Dan Shen	*Salvia miltiorrhiza Bunge*	Dried root and rhizome
*Syzygium aromaticum* (L.) Merr. & L. M. Perry.	Ding Xiang	*Caryophylli Flos*	Dried flower bud
*Cinnamomum camphora* (L.) J. Presl.	Bing Pian	*Borneolum Syntheticum*	Essential oil
Bambusa textilis McClure	Tian Zhu Huang	*Concretio Silicea Bambusae*	Dried mass of secretion

## Methods and Analyses

### Design and Settings

**FIGURE 1:**
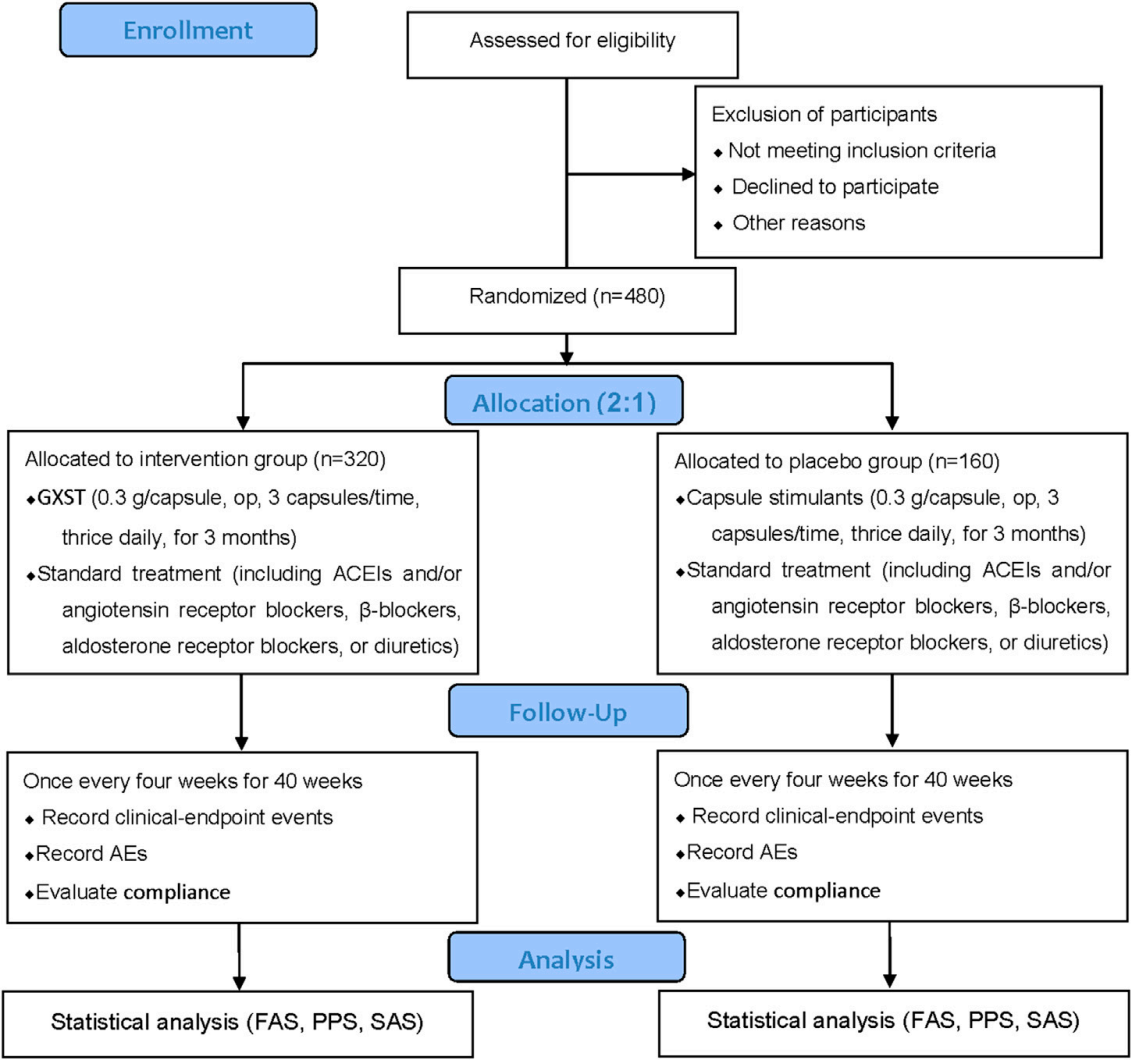
Flowchart of the clinical trial design. The template is from the CONSORT 2010 flowchart. GXST, Guanxinshutong; ACEI, angiotensin-converting enzyme inhibitor; AEs, adverse events; FAS, full analysis set; PPS, per protocol analysis set; SAS, safety assessment set.

This study is a multicenter, prospective, randomized, double-blind, placebo-controlled, superiority trial. The trial will be conducted by 11 centers throughout China (Table 2), and a total of 480 participants will be recruited. After the participants have been enrolled and provided written informed consent, they will be assigned randomly to the GXST group or placebo group in a 2:1 ratio. This trial consists of a 1-week baseline period, a 12-week intervention period, and a 40-week follow-up period. A flow diagram of the study procedures is illustrated in Figure 1. Participants will be monitored and assessed by the investigators at each study visit. The design follows the rules for Standard Protocol Items Recommendations for Interventional Trials (Chan et al., 2013) and Consolidated Standards of Reporting Trials (CONSORT) (Schulz et al., 2010).

**TABLE 2:** Research setting.

Second Affiliated Hospital of Zhejiang Chinese Medical University
The Affiliated Hospital of Hangzhou Normal University
Pingdingshan First People’s Hospital
Hangzhou First People's Hospital
Fenyang Hospital of Shanxi Province
The Affiliated Hospital of Liaoning University of Traditional Chinese Medicine
Tongxiang Traditional Chinese Medicine Hospital
Shaoxing People's Hospital
Pizhou Traditional Chinese Medicine Hospital
Lishui Central Hospital
First Affiliated Hospital of Xinxiang Medical University

### Recruitment

Recruitment strategies will include publishing recruitment advertisements on social media (e.g., QQ™, and We Chat™ in China, which are similar to Facebook™), online publications, and community centers. Patients who consent to participate will be examined and diagnosed by associate chief physicians to confirm their inclusion and will be registered on an online allocation system after written informed consent has been obtained.

### Study Population

The inclusion criteria and exclusion criteria are shown in Tables 3 and 4, respectively.

**TABLE 3:** Inclusion criteria.

Meet all the diagnosis of CHD (Chinese Society of Cardiology and Chinese Medical Doctor Association, 2018), CHF (Ponikowski et al., 2016; Heart Failure Group of Chinese Society of Cardiology and Editorial Board of Chinese Journal of Cardiology, 2018), and TCM diagnosis of blood stasis syndrome according to the *Guidelines for Clinical Research of New TCM Drugs* (Zheng, 2002)
Aged 40–80 years, of both sexes
New York Heart Association class II–III (Hunt et al., 2005)
Left ventricular ejection fraction <50% (using two-dimensional echocardiographic Simpson's method)
Clinical findings of CHF for ≥3 months before screening
Clinically stable in the last 1 month or receiving standardized treatment for ≥1 month, and no modification of dosage or intravenous administration has been given. Standardized treatment includes ACEIs and/or angiotensin receptor blockers, β-blockers, aldosterone receptor blockers, or diuretics
Plasma level of NT-proB-type natriuretic peptide ≥125 ng/L
Volunteer, understand, and provide written informed consent

**TABLE 4:** Exclusion criteria.

Acute coronary syndrome within the past 1 month
Plan to have cardiac surgery during the trial
Patients with cardiogenic shock, acute myocarditis, uncontrollable malignant arrhythmia, hypertrophic obstructive cardiomyopathy, pulmonary embolism, or severe valvular disease necessitating surgery
Uncontrolled hypertension, systolic blood pressure (SBP) ≥180 mmHg, and/or diastolic blood pressure (DBP) >110 mmHg; or SBP <90 mmHg and/or DBP <50 mmHg
Combined with severe liver or kidney dysfunction or active liver disease, and/or aspartate transaminase, alanine aminotransferase ≥3 times the upper limit of normal
Patients with diseases affecting walking ability, such as vascular disease of lower extremities
Combined with psychiatric manifestations
Patients with alcohol addiction or history of substance abuse
Females who are pregnant or lactating, preparing for pregnancy during the trial, or have a positive pregnancy test at the time of hospital admission
Have severe allergies, or allergic to research drugs and its ingredients
Patients with a history of previous or present malignancy, or precancerous lesions confirmed by pathology
Participation in other clinical studies within 3 months
Researchers estimate that the patient unable to complete the study

### Criteria for Withdrawal, Removal, Dropout, and Termination

The withdrawal criteria will be 1) exacerbation or deterioration that is clearly related to intake of the study drug; 2) an allergic reaction that is clearly associated with the study drug; 3) comorbidities, complications, adverse events (AEs), or serious adverse events (SAEs) during the trial, such as the necessity of long-term intravenous medication, repeated hospitalization for heart failure, or New York Heart Association (NYHA) class-IV symptoms; 4) the use of forbidden drugs or receipt of prohibited treatment affect the measures of efficacy and safety; 5) poor compliance by participants, or the amount of drug used does not meet the regulations (<80% or >120%); 6) participants request to withdraw from the study; 7) the blinding is uncovered or emergency unblinding is required.

Participants who fail to complete the observational period proposed in the trial, regardless of time and reasons, will be considered to be “dropout” cases. Reasons for dropout will be recorded in electronic case report forms (eCRFs), and the last data recorded will be included in data analyses. During the trial, a participant may be removed if he/she 1) is included mistakenly due to a misdiagnosis, 2) has not taken medication after study inclusion, 3) is participating in other clinical trials, and 4) has incomplete data and no evaluable records after inclusion. Another reason for removal is if the combined use of drugs has a great influence on the efficacy and safety of the study drug. Removal cases will not be included in analyses of the intention-to-treat (ITT) population.

The entire research study will be terminated if 1) there is a poor clinical effect of the test drug, and there is no need to continue the trial; 2) there are major defects in the trial; and 3) administrative authorities terminate the trial.

### Randomization, Allocation Concealment Mechanism, and Blinding

Participants will be randomized to the GXST group or placebo group in a 2:1 ratio using the Central Randomization System to achieve computerized randomization in blocks of six, stratified by center. The random sequence will be retained in an envelope and sealed subsequently. According to the randomization number, a statistician will send the envelope directly to Shaanxi Buchang Pharmaceutical Co., Ltd. for labeling of the intervention drug and placebo drug. All researchers, participants, physicians, drug administrators, and dispensing nurses will be blinded to the type of treatment until the study is completed. Unblinding will be available if participants experience an SAE or need to be rescued in an emergency situation. Once unblinded, the participant will withdraw from the study. Researchers should report the reasons to the inspector within 24 h. The precise cause of unblinding, date of the AE, the treatment situation, and the results must be recorded in eCRFs.

### Interventions

Eligible participants will be allocated randomly to the GXST group or placebo group. If GXST have been used before randomized, a 2-week drug washout period should be implemented to avoid any potential complication from GXST. All participants will receive standard treatment.

The GXST group will receive GXST (batch number: 190214, 0.3 g per capsule, op, 3 capsules per time, thrice daily). The placebo group will receive capsule stimulants (batch number: 190215, 0.3 g per capsule, op, 3 capsules per time, thrice daily).

Concerning standard treatment, during the intervention period, according to the guidelines for CHF treatment (Ponikowski et al., 2016; Chinese Society of Cardiology and Chinese Medical Doctor Association, 2018), ACEIs, and/or angiotensin receptor blockers, β-blockers, aldosterone receptor blockers, or diuretics should be used. TCM formulations with a similar composition and efficacy to those of GXST will not be allowed to be used. Concomitant treatments for comorbidities (e.g., hypertension, diabetes mellitus, hyperlipidemia, and other chronic conditions) are permitted during the intervention. Researchers should record the concomitant medication truthfully and maintain dose stability during the trial.

Concerning emergency treatment, in the event of an SAE or acute exacerbation of the disease during treatment (e.g., acute heart failure and acute coronary syndrome), participants should be treated first, and the treatment status should be recorded as an AE record form and combined medication record form. If the participant's condition deteriorates during treatment and it is not advisable to continue the trial (e.g., long-term intravenous medication, repeated heart failure that necessitates hospital admission, or NYHA class-IV symptoms), one should consider terminating the trial and switching to surgery or another type of clinical treatment. Patients will be classified in the analysis as “treatment ineffective.”

GXST and capsule simulants will be provided by Shaanxi Buchang Pharmaceutical Co. Ltd. The quality control of GXST is important. The method of determination of GXST components is based on the general principles of the 2018 edition of the *Chinese Pharmacopoeia*. Using gas chromatography (General Principle 0521), each GXST capsule should contain eugenol ≥2.0 mg and borneol ≥14.0 mg, and the total amount of borneol and isoborneol should be 25.5–34.5 mg. Using liquid chromatography (General Principle 0512), each GXST capsule should contain tanshinone II-A ≥0.15 mg and salvianolic acid B ≥2.9 mg. The primary content of capsule simulants is corn starch, silica, caramel (liquid), and sunset yellow. We have added 2% GXST powder to the capsule simulants to achieve smell, color, taste, and texture comparable with that of GXST. After treatment, the package will be returned to the researchers.

## Outcomes

### Primary and Secondary Outcomes

The details of items to be measured and the time window of data collection are shown in Table 5. The primary outcome is the improvement in 6-min walk distance (6MWD) (Holland et al., 2014). 6MWD will be measured within 24 h after enrollment and 12 weeks after treatment to evaluate the change in exercise tolerance. Improvement will be calculated using the following formula:

**TABLE 5:** Study schedule.

Study phase time	Baseline period	Intervention period			Follow-up
	Visit 1	Visit 2	Visit 3	Visit 4	Every 4 weeks
	−7 to 0 days	4 weeks	8 weeks	12 weeks	Until 52 weeks
Data collection at baseline					
Informed consent	×				
Inclusion/exclusion criteria	×				
Demographic data	×				
Obtain the central random number	×				
Previous history, medical history, and allergies	×				
Comorbidities and co-medications	×				
Safety evaluation					
Vital signs	×	×	×	×	
Physical examination	×	×	×	×	
Blood routine	×			×	
Urine routine	×			×	
Blood biochemistry	×			×	
Myocardial zymogram and troponin	×			×	
ECG	×			×	
Chest radiograph or CT	×			×	
Urine pregnancy test	×			×	
Efficiency evaluation					
6MWD	×			×	
NT-proBNP	×			×	
NYHA class	×	×	×	×	
MLHFQscore	×	×	×	×	
Echocardiographic parameters	×			×	
Clinical-endpoint events		×	×	×	×
Other work					
Dispense drug	×	×	×		
Recovery and record of study drug		×	×	×	
Metabolomics and proteomics	×			×	
Record AEs		×	×	×	×
Complications due to medications		×	×	×	
Evaluate compliance		×	×	×	×

CT, computed tomography; 6MWD, 6-min walk distance; NYHA, New York Heart Association; MLHFQ, Minnesota Living with Heart Failure Questionnaire; TCM, traditional Chinese medicine; AE, adverse event.

Improvement in exercise tolerance = 6MWD value after treatment − 6MWD value before treatment

The secondary outcomes are 1) changes in plasma levels of N-terminal pro-B-type natriuretic peptide (NT-proBNP) (Booth et al., 2014; Roberts et al., 2015). NT-proBNP will be detected centrally by Zhejiang Chinese Medical University using a standard kit and fixed operating procedures; 2) improvement in NYHA functional class (The Criteria Committee of the New York Heart Association, 1994); 3) improvement in the Minnesota Living with Heart Failure Questionnaire score (Rector and Cohn, 1992); 4) improvement in echocardiographic measurements of left ventricular end-diastolic diameter and LVEF; 5) the incidence rate of clinical endpoint events (rehospitalization for acute aggravation of CHF, cardiogenic death, and all-cause death).

### Safety Outcomes

Safety outcomes comprise vital signs (body temperature, heart rate, breathing, and blood pressure), imaging (radiographs or computed tomography), laboratory examinations (routine blood test, routine urinalysis, serum biochemistry, myocardial zymogram, and troponin test), and AEs (which will be recorded throughout the trial).

### Metabolomics and Proteomics Analyses

Fifty participants per group will be selected randomly for metabolomics and proteomics analyses to explore the biomarkers of GXST for treating CHF.

Metabolomics analyses will be on blood and urine samples. Gas chromatography–mass spectrometry and liquid chromatography–mass spectrometry will be used to detect chemical and biological “fingerprints,” describe the possible metabolic pathways, and identify biomarkers of GXST for CHF treatment.

Proteomics analyses will be of blood samples. Two-dimensional difference gel electrophoresis will be used to establish the proteome of each group before and after the intervention. Matrix-assisted laser desorption/ionization time-of-flight/time-of-flight mass spectrometry will be applied to identify differentially expressed proteins. Bioinformatic analyses are used to assess the biological functions of differentially expressed proteins. The Search Tool for the Retrieval of Interacting Genes/Proteins database and Ingenuity Pathway Analysis (IPA) are employed to establish a protein network affected by GXST.

Concerning the collection of blood samples, participants will fast for 10 h before each collection. Then, 5 ml of blood will be drawn and centrifuged, and serum stored in Eppendorf™ tubes at −70°C. Concerning the collection of urine samples, participants will fast for 10 h before taking the medication (and be allowed to drink water). They will fast, and water consumption will be prohibited 1 h before urine collection to 2 h after taking the medication. Water will be rationed to 200 ml/h for 2–8 h after taking the medication, and a low-fat meal can be consumed 4 h after taking the medication. Urine samples will be collected at baseline and after treatment (28 days) and stored in Eppendorf tubes at −70°C.

### Collection and Management of Data

According to the requirements of Good Clinical Practice and our research plan, the investigator shall input data to eCRFs accurately, completely, normatively, and in a timely fashion based on the original observations of participants. To ensure the accuracy of the data, two personnel specializing in data entry should undertake double-entry and proofreading independently. The auditor shall monitor whether the research is conducted following the research plan, ascertain whether all eCRFs have been completed correctly and are consistent with the original data, and issue questions at any time in case of any problem. If errors and omissions are made, the researcher shall be corrected promptly.

After inspection by the auditor, eCRFs must be transmitted to the department of data management in time. In this study, data management will be carried out with an electronic data-capture system by the Department of Medical Statistics within the First Hospital of Peking University (Beijing, China). The data administrator shall adopt system-automatic and manual-logic verification to check the consistency of eCRF data and source data. For questions in the eCRFs, the data administrator will generate a question–answer form and send an inquiry to the investigator through the clinical monitor. The investigator should answer the question as soon as possible, and the data administrator will modify the data according to the investigator's answer. If necessary, a question–answer form can be issued again. After being reviewed and confirmed by the primary researcher, sponsor, statistical analyst, and data administrator, the data will be locked and submitted to the statistician for analyses.

Personal information about potential and enrolled participants will be protected confidentially throughout the trial, and researchers should maintain data secrecy for five years after termination of the trial.

### Sample Size

The formula for calculating the sample size is based on the superior clinical trial sample size estimation (Wan et al., 2017). The sample size is driven by the expected improvement in 6MWD. Referring to clinical studies (Fan and Zhang, 2012; Guo and Li, 2013; Du, 2014; Wu et al., 2015), we assumed that the improvement in 6MWD is 30 m, and the combined standard deviation (SD) is 100 m in the present study. Given a rate of type-I error of *α* = 0.025, a power of 80% (rate of type-II error of *β* = 0.2), and, considering a possible dropout rate of 20%, 470 patients will need to be allocated. For the convenience of randomization, the final sample size was 320 cases in the intervention group and 160 cases in the placebo group, a total of 480 cases.

$$n_{1}={\left[\frac{\left({µ}_{\alpha }+{µ}_{\beta }\right)\sigma }{\delta }\right]}^{2}\frac{\left(1+k\right)}{k},\;n_{2}=k\;n_{1}.$$

In this formula, *k* is the ratio between two sample cases, *δ* is the expected improvement in 6MWD, and *σ* is the combined SD.

### Trial Completion

The trial will end once 480 patients have been randomized and all patients have completed 52 weeks of treatment and follow-up.

### Statistical Analyses

The statistical analysis plan will be specified before data analyses. The statistical analyses will be undertaken *via* Statistical Analysis System v9.4 (or higher version) by the Department of Medical Statistics, Peking University First Hospital. Professional statisticians who are independent of all other processes of our study will carry out analyses. Consistent with the CONSORT statement and ITT principle, and the last observation, carried forward method will be used for missing values. Cases in the per protocol set (PPS) will be those who adhere to the protocol closely without the absence of baseline characteristics. Analyses of primary outcome and curative effect will be carried out using a full-analysis-set approach and PPS approach. The safety analysis set will include all randomized patients who have accomplished at least one study visit. Participating centers will be required to sum-up participant numbers in each center and list participants who have been removed from PPS.

For continuous variables, we will calculate the mean, SD, median, minimum, maximum, and interquartile range. For categorical variables, we will describe various frequencies or percentages. The chi-square test or Fisher's exact test will be used for categorical variables. The Student's *t*-test will be used for continuous variables with a normal distribution. For data that do not have a normal distribution, intragroup or intergroup differences before and after treatment will be analyzed by the Wilcoxon rank-sum test. The proportion of patients with AEs in two groups will be compared using the chi-square test or Fisher's exact test. Because patients who had use of ARNIs before the recruitment may constitute a specific population, we will run sensitivity analyses of the outcomes with patients used ARNIs as basic treatment, and adjusted for the effect of ARNIs. Significance will be assumed at a two-sided *p*-value less than 5%. The relative risk with corresponding 95% confidence interval to compare dichotomous variables will be calculated. Table 6 shows the method of analysis for specific outcomes.

**TABLE 6:** Outcomes and methods of analyses.

Outcome/variable	Hypothesis	Measures	Methods of analyses
Baseline balance test			
		Quantitative outcomes (age, temperature, heart rate, respiratory rate, and blood pressure)	*t*-test/Wilcoxon rank-sum test
		Qualitative outcomes (sex, marriage, and previous treatment)	Chi-squared test/Fisher’s exact test/rank-sum test
Adherence at post-intervention		Percent and cases of adherence <80% and ≥80%	Chi-squared test/Fisher’s exact test
Concomitant treatments		Percent and cases of concomitant treatments	Chi-squared test/Fisher’s exact test
Primary outcome			
6MWD	Improvement occurred		*t*-test/Wilcoxon rank-sum test
			Covariance analysis
Secondary outcomes			
NT-proBNP	Improvement occurred		*t*-test/Wilcoxon rank-sum test
NYHA classification	Improvement occurred		Wilcoxon rank-sum test
MLHFQ	Improvement occurred	Questionnaire	*t*-test/Wilcoxon rank-sum test
Echocardiographic parameters	Improvement occurred	Left ventricular end-diastolic diameter and LVEF	*t*-test/Wilcoxon rank-sum test
Clinical endpoint events	Improvement occurred	Clinical-endpoint event rate	Log-rank test
Safety outcomes			
AEs, SAE		Percent and cases of AEs and SAEs	Chi-squared test/Fisher’s exact test
Vital signs		Change value relative to baseline	*t*-test/Wilcoxon rank-sum test

6MWD, 6-min walk distance; NT-proBNP: N-terminal pro-B-type natriuretic peptide; NYHA, New York Heart Association; MLHFQ, Minnesota Living with Heart Failure Questionnaire; LVEF: left ventricular ejection fraction; AE, adverse event; SAE: serious adverse events.

### Adverse Events

AEs that occur during the observation period will be monitored, reported to a research assistant, causality with the intervention will be evaluated, and the severity of AEs will be analyzed. SAEs will be reported to the ethics committee within 24 h. The classification and coding of AEs are formulated concerning Common Terminology Criteria for AEs version 4.03.

### Quality Control of the Intervention

To further ensure the quality of this trial, a multicenter trial coordination committee and general director will be set up for the implementation and solution of the problems related to this trial. The leaders of each center and the sponsor are the members of the coordination committee. Before each center recruits subjects, the sponsor will conduct unified preclinical trial training for all staff. Through such training, all staff (operators, investigators, physicians, data collectors, and analyzers) will fully understand the purpose and content of the trial, and standardize the process of data collection and CRF completion. This trial will be inspected by the CFDA, sponsor, and clinical research organization throughout the trial.

### Trial Status

This is an ongoing trial. The first participant was recruited on September 16, 2019. This study is recruiting participants currently.

## Discussion

CHF continues to be a major cause of mortality, initial and recurrent hospitalizations, and suboptimal quality of life (Yao et al., 2020). Despite advances in management and treatment of CHF, many CHF patients continue to suffer from exercise intolerance and dyspnea (Simpson et al., 2015). An increasing volume of evidence demonstrates that the combination of TCM with Western medicine could be an optimal approach for achieving greater treatment efficacy in patients with CHF (Gao et al., 2017; Jia et al., 2020).

GXST is a widely used TCM formulation for the treatment of cardiovascular disease in China. Pharmacology results have shown the effects of GXST in multiple mechanism pathways to treat CHF, such as improving hemorheology (Liang et al., 2012); enhancing mitochondrial capacity, and improving myocardial energy metabolism (Zhang et al., 2015; Zhang et al., 2016); and reversing ventricular remodeling (Zhang et al., 2012; Zhang et al., 2013). However, whether GXST is efficacious in patients with CHF requires confirmation by large-sample, multicenter, randomized controlled clinical trials. Therefore, we designed a multicenter, double-blinded, placebo-controlled clinical trial with the hope of verifying the efficacy and safety of GXST for the treatment of CHF with reduced LVEF. The results could provide a strategy for combining TCM and Western medicine in CHF patients.

We chose the 6MWD test to assess the functional capacity and exercise tolerance in patients with CHF. The 6MWD test is a simple and convenient test that requires no special equipment or advanced training for physicians and is well tolerated by patients. Although the plasma level of NT-proBNP is used widely in the differential diagnosis, risk stratification, and prognostic evaluation of CHF, use of ARNIs can affect the NT-proBNP level significantly (Yancy et al., 2017). Therefore, we chose the plasma level of NT-proBNP as a secondary outcome, and use sensitivity analyses to adjust the effect of ARNI on the outcomes. Furthermore, we have applied metabolomics and proteomics analyses to explore the therapeutic biomarkers of GXST in treating CHF, and to improve CHF therapy by providing an objective basis for precise treatment.

Our trial has three main limitations. First, this trial will be conducted in five provinces of China, and whether similar effects are obtainable in other regions and ethnic groups are unknown. Second, the treatment period will be 12 weeks with 40 weeks of follow-up, which is relatively short. Due to the limited time frame, the potential roles of GXST in reducing overall mortality and major vascular events over the long-term are uncertain, and further data on long-term clinical effect and safety will be needed. Third, GXST used in this trial is designed for the treatment of CHF with blood stasis syndrome, so the findings may not apply to other CHF syndromes.

### Ethics and Dissemination

The Research Ethics Committee of the Second Affiliated Hospital of Zhejiang Chinese Medical University (Hangzhou, China) has approved the study protocol (2019-Y-003-02). All participating centers have received study approval from their local ethics committee. All participants will provide voluntary written informed consent after a full discussion about the potential benefits and risks before participation. This study will be published in scientific journals to target a wide range of groups, and presented at national conferences in the field of CHF. Study results will also be sent to study participants and disseminated to researchers, as well as the general public through courses, presentations, and the Internet, regardless of the magnitude or direction of influence.

## Ethics Statement

The Research Ethics Committee of the Second Affiliated Hospital of Zhejiang Chinese Medical University has approved this study (No. 2019-Y-003-02). The patients/participants provided their written informed consent to participate in this study.

## Author Contributions

HW, SH, and FW designed the study. YW and JX contributed equally to the study, and conceptualized the study design and wrote the manuscript. LZ and JY modified the manuscript. YP, YH, and CL are responsible for the quality control of the test drug. LD, YX, JC, HZ, and LY participated in the modification of the study protocol. PZ and JY designed the method for statistical analyses. All authors read and approved the final version of the manuscript.

## Funding

This project is supported by the National Key R&D Program of China (2017YFC1700400, 2017YFC1700403) and the National Natural Science Foundation of China (81630105).

## Conflict of Interest

The authors declare that the research was conducted in the absence of any commercial or financial relationships that could be construed as a potential conflict of interest.

## Abbreviations

ARNI: angiotensin receptor neprilysin inhibitor; CHF: chronic heart failure; TCM: traditional Chinese medicine; CHD: coronary heart disease; GXST: Guanxinshutong; LVEF: left ventricular ejection fraction; ACEI: angiotensin-converting enzyme inhibitor; CFDA: China Food and Drug Administration; NT‐proBNP: N‐terminal pro-B-type natriuretic peptide; CONSORT: Consolidated Standards of Reporting Trials; FAS, full analysis set; PPS, per protocol analysis set; NYHA: New York Heart Association; eCRF: electronic case report forms; AEs: adverse events; SAE: serious adverse events; 6MWD: 6 min walk distance; SD: standard deviation; SAP: statistical analysis plan; ITT: intention to treat.
