# Supplementary material for: Effects of Guanxinshutong Capsules as Complementary Treatment in Patients With Chronic Heart Failure: Study Protocol for a Randomized Controlled Trial
Source: Front Pharmacol. 2021 Jan 14;11:571106. doi: 10.3389/fphar.2020.571106 (PMC7840487; doi:10.3389/fphar.2020.571106)
Supplement: Supplementary file 1 [file datasheet1.pdf]

Supplementary materials

Sample Collection, Transportation and Storage

1. Types of samples collection

In this trial, the subject's upper limb venous whole blood is collected, and the plasma is separated for the determination of NT-proBNP concentration.

2. Anticoagulant for sample collection

A vacuum blood collection tube containing EDTA-K2 anticoagulant was used to collect whole blood from the upper limb vein of the subject.

3. Label pattern of blood collection tube and test tube

Before blood sample collection, uniformly number blood collection tubes and cryopreservation tubes used for plasma sample testing and backup, and paste special labels. The label pattern of blood collection tubes and cryopreservation tubes are as follows:

Content	Form	Paraphrasing
Name	ABCD	Abbreviation of Subject Name
Subject	01-001	Center Number-Subject Random Number
Plasma	A	A is the test tube and B is the backup tube.
Visit	1	Visit times
Data	2018-09-23	Collection date

4. Storage conditions

After blood collection, centrifugation should be carried out within 1 hour, stored at -20℃for no more than 2 hours, and stored at -70℃ within 24 hours. The longest storage time of samples should not exceed 6 months.

5. Handover

Step 1: According to the conditions of sample preservation, CRO collect samples from each center and transport them to the central laboratory.

Step 2: Fill in the Sample Handover Registration Form before transportation. Record

the center number, abbreviation of the name of subject, drug code, testing items, number of visits, time and date of handover, quality of samples, signature of the submitter and recipient. If it was found that the sample does not meet the requirements (damaged tube, abnormal color, not enough volume, incorrect label, etc.), it should be rejected or re-processed.

Step 3: During transportation, all samples, Sample Handover Registration Form, thermometer and Biological Sample Transport Record Sheet are put in plastic bags, sealed with plastic tape, placed in plastic foam box, and filled with dry ice in the box to keep the temperature inside the box at -80℃. Then use tape to seal the foam box. It is strictly forbidden to invert the box during transportation. Only when the sample transport form is completed and signed by both parties can it be shipped out. Samples should be sent to the central laboratory within 12 hours (field research centers should be sent to the central laboratory within 24 hours).

Step 4: When the samples are sent to the central laboratory, the acceptor should check the samples, temperature and quality of the samples. Record the date and time of receipt, unpacking temperature, and quality of the sample on the "Sample Hand over Registration Form". If there are unqualified samples, inform the person in charge of the test immediately.

Step 5: When the samples are sent to the central laboratory, if they were not freeze-thawing, they should be stored inrefrigerator at -70℃ immediately; if they had been freeze-thawed, they should be stored inrefrigerator for refrigeration. Record sample storage temperature, time and date. Samples placed at 2-8℃ must be tested within 24 hours.

样品采集、运输与储存规范

1、生物样品的种类及确定依据

本试验拟采集受试者的上肢静脉全血，分离血浆用于进行NT-proBNP浓度测定。

2、样品采集拟采用样品管的抗凝剂

使用含 EDTA-K2 抗凝剂的真空采血管采集受试者的上肢静脉全血。

3、采血管及测试管的标签式样

在血液样品采集前对采血管及用于血浆样品测试与备份的冻存管进行统一编号，并粘贴专用标签。采血管及冻存管标签式样如下所示：

内容	样式	释义
Name	ABCD	受试者姓名缩写
Subject	01-001	研究中心编号-受试者随机号
Plasma	A	A 为分析测试管，B 为备份管
Visit	1	第几访视
Data	2018-09-23	样本采集日期

4、标本存放条件

采血后应在 1h 内进行离心，-20℃保存不超过 2h，24h 内转入-70℃保存，每个样品储存时间最长为 6 个月。

5、样本交接

第一步：根据样本保存条件，由 CRO 工作人员从各中心收集样本，运往中心实验室。

第二步：运输前填写《样本交接登记表》。记录研究中心编号、受试者姓名缩写、药物编码、检测项目、访视次数、工作人员交接时间和日期、注明样本质量、递交人及接收人签字。如发现样本不符合要求（管破、颜色异常、量不够、标签有误等），应拒绝或再处理接收。

第三步：运输时将所有样本、填好的《样本交接登记表》及温度计和《生物样本运输记录单》放入塑料袋内，用胶布封严，置于塑料泡沫箱内，箱内充满干冰，使箱内温度尽量保持在-80℃。再用胶带封严泡沫塑料箱。运输过程中箱体严谨倒置。填写样本运输单，双方签字，方可运出。样本要求 12 小时内送至中心实

验室（外地研究中心需在 24 小时内送至中心实验室）。

第四步：各研究中心的样本送至中心实验室时，寄售人员核对送检样本，检查样本温度及质量。在《样本交接登记表》上记录接收日期和时间、开箱温度、样本质量并签名；如有不合格的样本，立即通知试验负责人。

第五步：各研究中心的样本送至中心实验室时，如未冻融，立即放-70℃冰箱保存；如已经冻融，放置冰箱冷藏。记录样本保存温度、时间、日期。在 2-8℃放置的样本必须在 24 小时内完成检验。
